# Mathematical Modeling of Oncogenesis Control in Mature T-Cell Populations

**DOI:** 10.3389/fimmu.2013.00380

**Published:** 2013-11-21

**Authors:** Sebastian Gerdes, Sebastian Newrzela, Ingmar Glauche, Dorothee von Laer, Martin-Leo Hansmann, Ingo Roeder

**Affiliations:** ^1^Institute for Medical Informatics and Biometry, Medical Faculty Carl Gustav Carus, Dresden, Germany; ^2^Senckenberg Institute of Pathology, Goethe-University Hospital Frankfurt, Frankfurt, Germany; ^3^Department of Hygiene, Medical University Innsbruck, Innsbruck, Austria

**Keywords:** T-cell homeostasis, T-cell niche, gene therapy, mature T-cell lymphoma, MTCL

## Abstract

T-cell receptor (TCR) polyclonal mature T cells are surprisingly resistant to oncogenic transformation after retroviral insertion of T-cell oncogenes. In a mouse model, it has been shown that mature T-cell lymphoma/leukemia (MTCLL) is not induced upon transplantation of mature, TCR polyclonal wild-type (WT) T cells, transduced with gammaretroviral vectors encoding potent T-cell oncogenes, into RAG1-deficient recipients. However, further studies demonstrated that quasi-monoclonal T cells treated with the same protocol readily induced MTCLL in the recipient mice. It has been hypothesized that in the TCR polyclonal situation, outgrowth of preleukemic cells and subsequent conversion to overt malignancy is suppressed through regulation of clonal abundances on a per-clone basis due to interactions between TCRs and self-peptide-MHC-complexes (spMHCs), while these mechanisms fail in the quasi-monoclonal situation. To quantitatively study this hypothesis, we applied a mathematical modeling approach. In particular, we developed a novel ordinary differential equation model of T-cell homeostasis, in which T-cell fate depends on spMHC-TCR-interaction-triggered stimulatory signals from antigen-presenting cells (APCs). Based on our mathematical modeling approach, we identified parameter configurations of our model, which consistently explain the observed phenomena. Our results suggest that the preleukemic cells are less competent than healthy competitor cells in acquiring survival stimuli from APCs, but that proliferation of these preleukemic cells is less dependent on survival stimuli from APCs. These predictions now call for experimental validation.

## 1 Introduction

Mature T cells are an essential component of the adaptive immune system. They carry the so-called *T-cell receptor* (TCR) on their surface. This receptor enables them to recognize peptides that are presented to them via major histocompatibility complex (MHC) molecules on antigen-presenting cells (APCs). A vast number of different TCRs is expressed in T cells in healthy individuals, estimated to be in the order of 10^6^ in mice (1) and 10^7^ in humans (2). An individual T cell expresses a single TCR variant, and passes this variant on to its daughter cells. The set of all T cells expressing the same TCR is called a *T-cell clone* (or simply referred to as *clone*). The enormous TCR diversity is created during T-cell maturation in the thymus through genomic rearrangement of the TCR gene locus (3). A series of selection processes ensures that mature T cells can bind with low to moderate affinity to self-peptide-MHC complexes (spMHCs) on APCs (4–6). After maturation, T cells enter the peripheral T-cell pool.

The peripheral T-cell pool is remarkably stable in terms of cell numbers and clonal diversity throughout the lifetime of mice and humans. In order to explain this stability, concepts have emerged that are based on competition between T cells for limiting trophic resources needed for survival and proliferation (7). The limiting trophic resources can be divided in public and TCR-specific resources (8). In principle, public trophic resources are equally accessible to all T cells, and include stimulatory cytokines (e.g. interleukin 7), nutrients, costimulatory molecules, or physical space. In contrast, access to TCR-specific resources depends on the particular TCR that is expressed on a T cell. TCR-specific resources are represented mainly by stimulatory interactions with APCs due to binding of the TCR to spMHCs (9–11).

Different spMHCs may vary substantially in their suitability to mediate a stimulatory interaction for particular T-cell clones. Consequently, a *T-cell niche* concept has been proposed, in which different spMHCs represent distinct T-cell niches (12). The niches provide vital resources that different T-cell clones compete for. A particular clone may not receive resources from all niches equally well. This concept implies that the TCR diversity is stabilized by the diversity of the available spMHCs (13).

When the regulation of cellular proliferation in the T-cell system is corrupted, mature T-cell lymphoma/leukemia (MTCLL) formation may occur. However, oncogenesis is comparatively rare in mature T cells. For example, the incidence of B-cell lymphoid neoplasms is substantially higher than the incidence of T-cell/natural killer cell lymphoid neoplasms, as shown in a study from the United States (26.13/10^5^/year vs. 1.79/10^5^/year (14)). Furthermore, several studies from the field of retroviral gene therapy confirm the relative resistance of mature T cells to oncogenesis. Despite long follow-up times, retroviral vector-induced oncogenesis has never been observed in clinical gene therapy trials involving gene-modified mature T cells (15–17). In contrast, genotoxicity was observed in several studies involving retroviral gene transfer into hematopoietic stem and progenitor cells (HSPCs) (18, 19).

Motivated by these observations, we here focus on the analysis of oncogenesis control in mature T-cell populations.

In order to explicitly investigate the relative resistance of mature T cells to malignant transformation in a gene therapeutic context, HSPCS, and mature T cells were exposed to an identical transformation assay in a defined experimental setting (20). In this assay, HSPCs and mature T cells were isolated from wild-type mice and were each transduced independently with high copy numbers of gammaretroviral vectors encoding potent T-cell oncogenes. Subsequently, the cells were transplanted into immunoincompetent RAG1-deficient mice. HSPC-transplanted animals consistently developed MTCLL. In contrast, MTCLL has not been observed in any of the recipients that were transplanted with mature T cells. This finding corroborated the relative resistance of mature T cells to malignant transformation.

**Figure 1:**
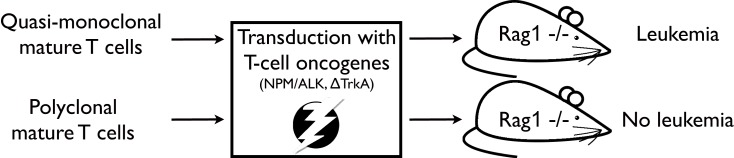
**Experimental strategy as described in Newrzela et al. (21)**. TCR quasi-monoclonal T-cell populations transduced with potent T-cell oncogenes developed mature T-cell lymphoma/leukemia in RAG1-deficient recipient mice, while TCR polyclonal T-cell populations that have been subjected to the same transformation assay did not.

In a subsequent study, the impact of TCR diversity on T-cell resistance to malignant transformation has been further assessed (21). In this study, T-cell populations were isolated from OT1- or P14-mice, i.e. mice expressing a transgenic TCR. T-cell populations from these mouse models are quasi-monoclonal, i.e. they express predominantly one specific TCR. By applying a similar, yet refined, transformation assay as in the previous study MTCLL readily developed in the recipient RAG1-deficient mice (see Figure 1). Moreover, addition of untransduced TCR polyclonal T cells to quasi-monoclonal, transduced cell populations prevented malignancy development, demonstrating that TCR polyclonality plays a pivotal role in malignancy control in mature T cells.

Building on these observations, we hypothesize that in the TCR polyclonal situation, prohomeostatic signals, due to interactions between TCRs and spMHCs, suppress the outgrowth of preleukemic T cells (i.e. in this context, T cells that have been transformed by retroviral insertion of an oncogene), while these mechanisms fail in the TCR quasi-monoclonal situation, and vigorous cell expansion occurs. In this paper, we aim to quantitatively assess the implications of this hypothesis using a mathematical modeling approach. Specifically, we develop a mathematical model of a niche-dependent mature T-cell regulation [similar to previous published models, e.g. Ref. (22)], which will be applied to model the physiological situation and MTCLL formation. Using this model of T-cell homeostasis, we present an *in silico* simulation scenario that is suited to mimic the experimental procedures performed by Newrzela et al. (20, 21). In an extensive parameter screen, we evaluate if, and under which parameter constellations the experimental observations can be explained.

## 2 Materials and Methods

### 2.1 Model description

The two major entities in our model are *T-cell species* (also called simply *species*) and *T-cell niches* (also called simply *niches*). With the term *species*, we refer to a set of T cells that is homogeneous in terms of our model parameters. In the physiological situation, a T-cell clone can be represented as a particular species. However, in order to model the aforementioned experimental situation (20, 21), we will represent each T-cell clone by two species, namely a species representing healthy cells within a clone and a preleukemic species representing cells that potentially give rise to MTCLL.

Our model is constructed to describe the temporal dynamics of species abundances, i.e. the number of cells belonging to a particular T-cell species at any point of time. The number of species in the system is denoted by the symbol *m*. The species abundances at time *t* are represented by the vector **c**(*t*) = (*c_t_*(*t*)) with *i* = 1, 2, …, *m*. The initial species abundances **c**(0) are defined by an *m*-dimensional vector denoted $c^{0}=\left(c_{i}^{0}\right).$

In the model, T-cell species compete for resources needed for survival and proliferation that are supplied by T-cell niches. The number of niches in the system is denoted by the symbol *n*. It is assumed that the niches supply resources at constant rates, represented by the *n*-dimensional vector **p** = (*p_j_*), where *j* = 1, 2, …, *n*.

The competition for niche resources between species is defined by the *m* × *n* matrix **A** = [*a_ij_*], called *niche affinity matrix*, in which *a_ij_* is a measure of the capability of species *i* to acquire resources from niche *j*.

At any time point *t*, species receive resources from niches according to instantaneous rates. These rates, which are termed *resource acquisition rates*, are stored in an *m* × *n* matrix **R**(*t*) = [*r_ij_*(*t*)]. *r_ij_*(*t*) denotes the rate at which the *i*-th species receives resources from the *j*-th niche at time *t*, and is the affinity- and abundance-weighted proportion of the total rate *p_j_* at which niche *j* provides resources. Thus, *r_ij_*(*t*) is calculated as follows:

$$\begin{array}{ll} r_{ij}\left(t\right) & =\left\{\begin{array}{cc} \frac{a_{ij}c_{i}\left(t\right)}{\sum_{k=1}^{m}\;a_{kj}c_{k}\left(t\right)}p_{j}\; & \mathrm{if}\sum_{k=1}^{m}\;a_{kj}c_{k}\left(t\right)>0\\ 0\; & \mathrm{otherwise} \end{array}\right. \end{array}$$

The sum of the resource acquisition rates over all niches yields the net resource acquisition rate for a particular species.

Different T-cell species may differ in the amount of resources needed to sustain an individual cell. Therefore, we introduce the *m*-dimensional vector **v** = (*v_i_*), which we term *resource utilization efficiency*. This parameter determines for each species, how many T cells can be sustained by one resource unit per unit of time. The product of the resource acquisition rates and the resource utilization efficiencies yields the time-dependent carrying capacities of the individual species, stored in the vector **k**(*t*) = (*k_i_*(*t*)):

$$k_{i}\left(t\right)=v_{i}\sum_{j=1}^{n}\;r_{ij}\left(t\right)$$

The carrying capacities indicate how many cells of a given species can be sustained by the system, given the current resource distribution among the species. The carrying capacities may change dynamically in time, as the species abundances vary. The relation between the actual size of a species and the carrying capacity determines whether the species abundance decreases or increases. Due to lack of biological data determining a particular growth model for T-cell clones, we chose a rather general logistic growth dynamics, where τ denotes the minimum cell cycle time:

$$\frac{dc_{i}\left(t\right)}{dt}=\left\{\begin{array}{cc} \frac{c_{i}\left(t\right)}{\tau }\left(1-\frac{c_{i}\left(t\right)}{k_{i}\left(t\right)}\right)\; & \mathrm{if}k_{i}\left(t\right)>0\\ 0\; & \mathrm{if}k_{i}\left(t\right)=0\\ \; \end{array}\right.$$

We require $c_{i}^{0}$, *a_ij_*, *p_j_*, and *v_i_* to be greater than or equal to zero, and τ to be greater than zero.

### 2.2 Parameter choice

In its general form, the model presented in the previous subsection has *nm* + 2*m* + *n* + 1 scalar parameters. In the following, we sketch our numerical approach and reparameterize the model in order to reduce the effective number of parameters.

In our approach, the number of TCR-defined clones is denoted by *q*. Each clone is represented by two species (thus *m* = 2*q*). Without loss of generality, species 1 to *q* represent the presumably healthy cells within the *q* clones (referred to as *healthy species*) and species *q* + 1 to 2*q* represent the cells that potentially give rise to leukemia/lymphoma (referred to as *preleukemic species*). The specific case of the monoclonal situation is represented by setting all but the first healthy and the first preleukemic initial species abundances to zero (*c_i_* = 0 except *c*_1_ > 0, *c_q_*_ + 1_ > 0). We assume that each species may in principle receive a stimulus from each niche due to unspecific affinity. The magnitude of the unspecific affinity is denoted *u*^(*h*)^ for the healthy species and *u*^(^*^p^*^)^ for the preleukemic species. In addition, we assume that each species has a preferred niche, to which it has an additional affinity [*specific affinity*, denoted *s*^(*h*)^ for the healthy species and *s*^(^*^p^*^)^ for the preleukemic species]. Biologically, the specific affinity could be interpreted as affinity to cognate self-peptide, and the unspecific affinity as affinity to the MHC itself.

The scalar parameters describing the specific and the unspecific affinities are used to define the niche affinity matrix **A**. Formally, we construct the matrices $A^{\left(h\right)}=\left[a_{ij}^{\left(h\right)}\right]$ describing the niche affinities of the healthy species and $A^{\left(p\right)}=\left[a_{ij}^{\left(p\right)}\right]$ describing the niche affinities of the preleukemic species as follows,

$$\begin{array}{llll} & a_{ij}^{\left(h\right)}=\left\{\begin{array}{cc} s^{\left(h\right)}+u^{\left(h\right)}\; & \mathrm{if}\;i=j\\ u^{\left(h\right)}\; & \mathrm{if}\;i\neq j\\ \; \end{array}\right.\; & & \;\\ & a_{ij}^{\left(p\right)}=\left\{\begin{array}{cc} s^{\left(p\right)}+u^{\left(p\right)}\; & \mathrm{if}\;i=j\\ u^{\left(p\right)}\; & \mathrm{if}\;i\neq j\\ \; \end{array}\right.\; & & \;\\ & i\in \left\{1,\;2,\;...,\;q\right\},\;\;j\in \left\{1,\;2,\;...,\;n\right\}\; & & \; \end{array}$$

and use these matrices in order to construct the actual niche affinity matrix **A** by vertical concatenation of **A**^(*h*)^ and **A**^(^*^p^*^)^:

$$A=\left[\begin{array}{c} A^{\left(h\right)}\\ A^{\left(p\right)} \end{array}\right]$$

Furthermore, we introduce the symbols *v*^(*h*)^ and *v*^(^*^p^*^)^ to describe the resource utilization efficiencies of the healthy and preleukemic species, respectively. *v*^(*h*)^ and *v*^(^*^p^*^)^ are used to construct the actual resource utilization efficiencies (superscript *T* denoting the transpose of a vector):

$$v={\left(\underset{q\;\mathrm{times}}{\underset{︸}{v^{\left(h\right)},\;\ldots ,\;v^{\left(h\right)}}},\;\underset{q\;\mathrm{times}}{\underset{︸}{v^{\left(p\right)},\;\ldots ,\;v^{\left(p\right)}}}\right)}^{T}$$

Note that no interspecies heterogeneity with respect to the magnitudes of the specific and unspecific affinities of the healthy species (*s*^(*h*)^, *u*^(*h*)^) and their resource utilization efficiency *v*^(*h*)^ is considered. The same applies to the preleukemic species, with parameters *s*^(^*^p^*^)^, *u*^(^*^p^*^)^, and *v*^(^*^p^*^)^.

In order to make numerical simulations feasible, while still preserving the niche-based regulation, we are using a system with 100 niches (*n* = 100) and 100 clones (*q* = 100), i.e. 200 species (*m* = 200) for the numerical simulations.

As mentioned above, the real number of T-cell clones in mice is estimated in the order of 10^6^, while the total number of T cells in a mouse is estimated to be in the order of 10^8^ (23). Assuming that in the physiological situation the number of niches in mice equals the number of T-cell clones, a niche nourishes 100 cells on average. Therefore, we consider the niche sizes *p_j_* = 100 for all *j* = 1, …, 100. The minimum cell cycle time τ is set to 8 h as a rough estimate of vigorous T-cell proliferation (24). Since an initial exploratory screen did not reveal a qualitative effect of changes in niche size **p** and the minimum cell cycle time τ on the model behavior, we keep **p** and τ fixed to the above described values.

Additionally, and without loss of generality, we can fix one of the four niche affinity parameters *s*^(*h*)^, *s*^(^*^p^*^)^, *u*^(*h*)^, and *u*^(^*^p^*^)^ as the distribution of resources depends on relative affinities only. We chose to fix the unspecific affinity of the healthy cells *u*^(*h*)^ = 1/*n* = 0.01 (*n* being the number of niches) as our reference. The specific affinity of the healthy species *s*^(*h*)^ is set to 1 [except for the results shown in Figure 6, in which we are also considering the values *s*^(*h*)^ = 0.2 and *s*^(*h*)^ = 5]. Furthermore, we set the resource utilization efficiency of the healthy cells *v*^(*h*)^ to 1, thus interpreting the corresponding value of the preleukemic cells *v*^(^*^p^*^)^ as a relative measure compared to the healthy situation.

Hence, at this point the scalar parameters *s*^(^*^p^*^)^ (specific affinity of the preleukemic species), *u*^(^*^p^*^)^ (unspecific affinity of the preleukemic species), *v*^(^*^p^*^)^ (resource utilization efficiency of the preleukemic species), and the vector-valued initial condition **c**^0^ are not yet fixed. How they are varied in the parameter screen is described in the following subsection.

### 2.3 Simulation procedure

#### 2.3.1 Physiological situation

In order to study the behavior of the system, we *in silico* transplant 500 cells into an empty system. TCR diversities *q* = 1 and *q* = 100 are considered. In the TCR polyclonal situation, the initial species abundances **c**^0^ are obtained by distributing the 500 cells according to a uniform distribution over the healthy species. *s*^(*h*)^ is set to 1. The parameters *s*^(^*^p^*^)^, *u*^(^*^p^*^)^, and *v*^(^*^p^*^)^, which describe the properties of the preleukemic species, do not influence the model behavior here, since no preleukemic cells are transplanted into the system. All other model parameters are fixed to the above described values. We let the system evolve for 400 time-steps. To demonstrate the system response to perturbations, we simulate a significant cell loss by removing 99% of the cells at time *t* = 200. The simulation results are presented in subsection 1.

#### 2.3.2 Oncogenic situation

In order to systematically evaluate the effect of the parameters describing the properties of the preleukemic cells [i.e. their specific affinity *s*^(^*^p^*^)^, their unspecific affinity *u*^(^*^p^*^)^, their resource utilization efficiency *v*^(^*^p^*^)^] and the initial abundances **c**^0^, we perform an extensive parameter screen. *s*^(^*^p^*^)^, *u*^(^*^p^*^)^, and *v*^(^*^p^*^)^ are varied in multiples of their healthy counterparts, ranging from ∼1/40 to ∼40. The relative distances between two neighboring values is 20%, so that 41 different fold-changes are considered per parameter.

The fraction of transplanted cells that have been transformed into preleukemic cells is currently not known for the used experimental protocol. Therefore, we consider three scenarios, denoted P1, P10, and P100, which consider 1, 10, and 100 initial preleukemic cells, respectively. All three scenarios are evaluated for each combination of the specific affinity of the preleukemic species *s*^(*p*)^, their unspecific affinity *u*^(*p*)^, and their resource utilization efficiency *v*^(*p*)^. All cases are initiated by 500 cells that are *in silico-transplanted* into an empty system.

This corresponds to 5 cells per clone in the polyclonal situation on average, in accordance with the experimental situation (5 × 10^6^ transplanted cells, TCR diversity estimated in the order of 10^6^). In scenario P1, one T cell (0.2%) is assigned to the preleukemic cell compartment, in scenario P10, 10 T cells (2%), belonging to 10 different species in the TCR polyclonal situation, are assigned to the preleukemic cell compartment, and in scenario P100, 100 T cells (20%), belonging to 100 different species in the polyclonal situation. In all three scenarios, the remaining cells are distributed randomly according to a uniform probability distribution over the healthy species in the polyclonal situation. In addition to the polyclonal settings we construct a corresponding monoclonal situation, in which the fraction of 0.2% (P1), 2% (P10), or 20% (P100) preleukemic cells is assigned to only a single clone.

The number of parameter sets evaluated in each of the three scenarios is 41^3^ (since 41 different fold-changes are considered for *s*^(^*^p^*^)^, *u*^(^*^p^*^)^, and *v*^(^*^p^*^)^). Hence, in total 3 × 41^3^ ≈ 2 × 10^5^ parameter sets are evaluated for both the polyclonal and the monoclonal situation.

As we are not primarily interested in transient phenomena but in the long-term behavior of the system, our aim is the identification and characterization of stable steady states, namely whether the preleukemic cells are able to dominate the system or not. Therefore, simulations are run until the relative change of all species abundances between two successive time steps are below 10^−6^. For this situation we assume that the system is sufficiently close to a steady state. If this criterion is not fulfilled after 10^8^ simulation steps, the current simulation is stopped, and the stability of the system will be assessed manually.

For each individual parameter set, we evaluate if the corresponding simulations for the mono- and polyclonal situation are consistent with the experimental phenomena, i.e. if we observe a stable and considerably enlarged population of preleukemic cells in the monoclonal situation, and control of the preleukemic cells in the polyclonal situation (i.e. the contribution of the preleukemic cell population remains below a certain threshold). The specific criteria used for the classification are listed in Table 1. If a parameter set fulfills all criteria, we consider it consistent with the observed phenomena as described in (20, 21).

**Table 1:** **Criteria used for classification**.

	Total cell count	Contribution of preleukemic cells
TCR monoclonal situation	At least 300% of physiological cell count	At least 80% of total cell count
TCR polyclonal situation	At most 120% of physiological cell count	At most 50% of total cell count

A particular tested parameter set is classified as consistent with the experimental phenomena, if the total cell count and the contribution of the preleukemic cells fulfill the criteria specified in the table, i.e. a leukemia-like situation in the TCR monoclonal situation, and a state of non-dominance of the preleukemic species in the TCR polyclonal situation. The total cell count at the steady state is compared to the physiological cell count, which is established based on the simulations shown in subsection 1. The contribution of the preleukemic cells is the ratio between the number of preleukemic cells and the total cell count at the steady state.

The simulation results of the parameter screen are presented in subsection 2.

## 3 Results

### 3.1 Physiological situation

**Figure 2:**
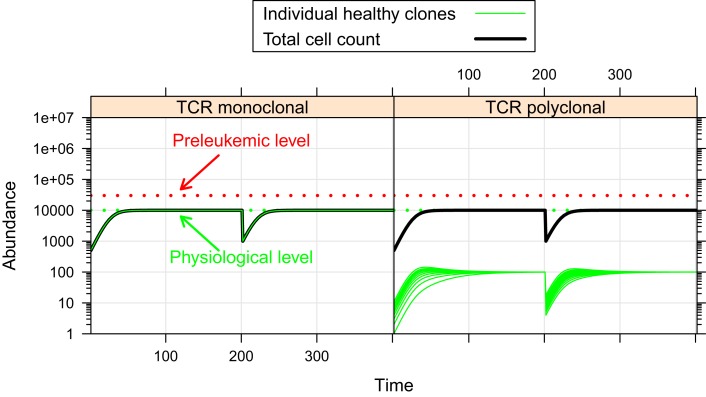
**Simulation results of physiological situation**. The left panel documents the dynamic system behavior in the monoclonal situation. In the right panel, the individual green lines represent the abundance of different clones. The system quickly converges to a steady state. The total cell count in the steady state of the physiological situation is indicated by the horizontal dashed green line at 10^4^. The horizontal red line at 3 × 10^4^ represents the total cell count that is required as a minimum in order to qualify a situation as premalignant. At *t* = 200, 99% of the cells are removed from the system. Both in the mono- and the polyclonal situation the system quickly reestablishes the previous steady state.

First, we investigate the behavior of the described system in the absence of preleukemic cells. As can be seen in Figure 2, the system quickly converges to a steady state, both in the mono- and the polyclonal situation. In the polyclonal situation, all clones have an abundance of 100 cells at the steady state due to the parameter symmetry among the healthy species (i.e. all species have the same specific affinity *s*^(*h*)^ = 1 to their preferred niche, and the same unspecific affinity *u*^(*h*)^ = 1/*n* to all niches). The total cell count at the steady state amounts to $v^{\left(h\right)}\cdot \sum_{j=1}^{n}\;p_{j}=10^{4}$ both in the monoclonal and the polyclonal situation. After a perturbation, e.g. due to cell kill, the system quickly reestablishes its previous state, unless individual species are completely eliminated within the cell kill simulation.

The steady state with equal-sized clones is reached regardless of the initial abundances of the healthy cells, given that the abundances of all healthy species are >0 (data not shown). Transient changes of the niche sizes or transient addition of niches (e.g. to model infections) can entail the transient expansion of one more species/clones (data not shown).

### 3.2 Oncogenic situation

**Figure 3:**
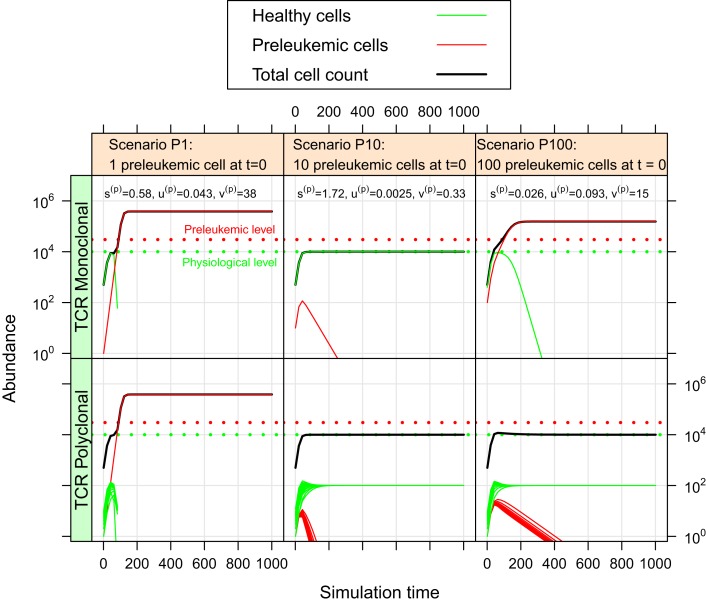
**Simulation results of three representative parameter sets**. For each scenario, we show the simulation results of one possible parameter set. The parameter set picked for scenario P1 is not consistent with the experimental observations as specified in Table 1. The preleukemic cells expand more than threefold both in the mono- and polyclonal situation. The parameter set chosen for P10 is also not consistent with the specified criteria. Using this parameter set, the preleukemic cells are less fit than the healthy cells, and die out both in the mono- and the polyclonal situation. The parameter set chosen for scenario P100 is consistent with the specified criteria.

For the oncogenic situation, we evaluate whether a certain proportion of preleukemic cells (represented by the three scenarios P1, P10, and P100) can develop into a (pre)leukemic situation in the monoclonal case, while being controlled by healthy competitor cells in the polyclonal case. The evaluation is carried out based on prespecified formal criteria (see Table 1). In all tested parameter settings, the steady state (i.e. convergence) criterion is reached both in the mono- and the polyclonal situation. Figure 3 shows representative simulation results to provide some intuition about the spectrum of possible simulation outcomes for the three scenarios P1, P10, and P100.

**Figure 4:**
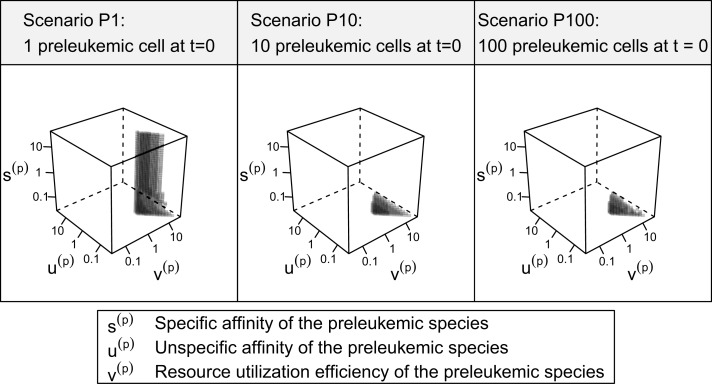
**3D scatter plot of consistent parameter sets**. Each tested parameter set can be represented by a point in one of the three presented cubes, the coordinates representing the fold-changes of *s*^(^*^p^*^)^, *t*^(^*^p^*^)^, and *v*^(^*^p^*^)^ in comparison with their healthy counterparts *s*^(*h*)^, *u*^(*h*)^, and *v*^(*h*)^. In the center of each cube, the parameters *s*^(^*^p^*^)^, *u*^(^*^p^*^)^, and *v*^(^*^p^*^)^ are equal to their counterparts describing the healthy cells. Only the parameter sets that are consistent with the experimental observations (criteria in Table 1) are plotted. Transparency is used in order to give an impression of the shape of the consistent parameter region.

Out of the ≈2 × 10^5^ tested parameter configurations, 1050 parameter sets are consistent with the experimental phenomena according to the defined criteria (c.f. Table 1). Further, it should be emphasized that the consistent parameter sets are identifiable as a confined region in the parameter space in all three scenarios (see Figure 4) and that the regions in scenarios P1, P10, and P100 overlap considerably. In all three scenarios, we identify a region that is characterized by lowered specific and unspecific affinities as well as an increased resources utilization of preleukemic compared to normal T cells. Technically, this refers to triangular prisms in the $u_{low}^{\left(p\right)}s_{low}^{\left(p\right)}v_{high}^{\left(p\right)}$ octant of the parameter cube (Figure 4). In scenario P1, the region of consistent parameter sets additionally extends to the $u_{low}^{\left(p\right)}s_{high}^{\left(p\right)}v_{high}^{\left(p\right)}$ octant. Our results indicate that the overall systems behavior displays only a minor dependency on the initial number of preleukemic cells with respect to the phenomena in focus.

**Figure 5:**
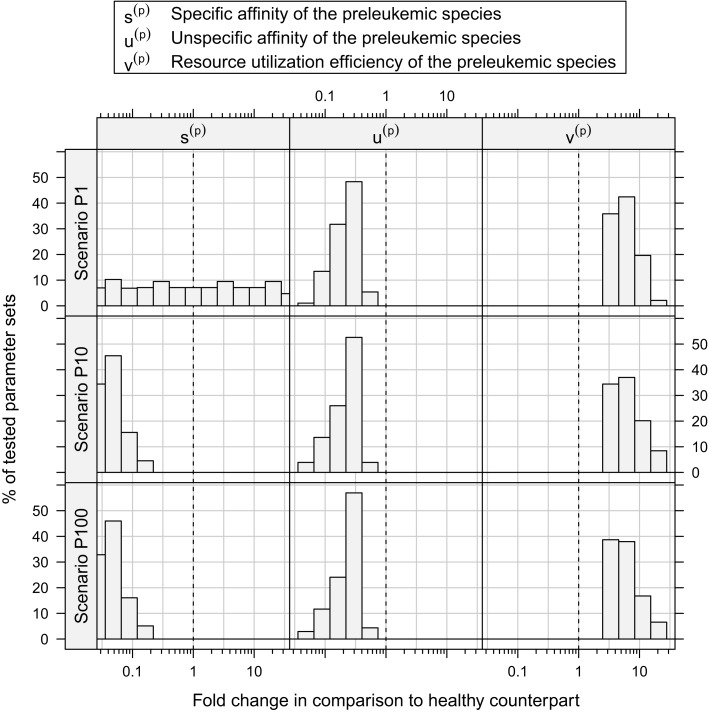
**Histograms of consistent parameter values**. These histograms show the distribution of the specific affinity of the preleukemic species *s*^(*p*)^, their unspecific affinity *u*^(*p*)^, and their resource utilization efficiency *v*^(*p*)^ of the consistent parameter sets. *s*^(*p*)^ is decreased in all consistent parameter sets of scenario P10 and P100, while it is indifferent in scenario P1. *u*^(*p*)^ is decreased and *v*^(*p*)^ is increased in all consistent parameter sets.

Assessing the set of consistent parameter constellations in more detail, we find that the resource utilization efficiency parameter for the preleukemic species *v*^(*p*)^ had to be chosen at least three-fold higher than the resource utilization efficiency for healthy cells, in order to be consistent with the experimental observations. This is more clearly seen in Figure 5, which further characterizes the parameter sets that are consistent with the predefined criteria. The fact that a three-fold increase of *v*^(*p*)^ is required, directly reflects criterion from Table 1, stating that the cell counts have to be increased at least three-fold in the monoclonal situation.

Concerning the niche affinities of the preleukemic cells, a clear pattern can be observed, likewise apparent in Figure 5. In scenarios P10 and P100, the specific affinity of the preleukemic cells *s*^(^*^p^*^)^ is decreased (i.e. *s*^(^*^p^*^)^ < *s*^(*h*)^) in all consistent parameter sets. In scenario P1, however, the specific affinity can be considerably increased without rendering a tested parameter set inconsistent. This is due to the fact that in scenario P1, only a single cell is preleukemic initially. Therefore, the fitness of the preleukemic cell pool (i.e. proliferative potential) increases only marginally in the polyclonal situation if the specific affinity of the preleukemic cells *s*^(*p*)^ is increased. In contrast, in scenario P10 and P100, the fitness of the preleukemic cell population in the polyclonal situation responds more sensitively to an increase of the specific affinity of the preleukemic cells due the greater TCR diversity within the preleukemic cell population. Because of that, the preleukemic cells cannot be controlled by healthy competitor cells in the polyclonal situation, even if the specific affinity is only mildly increased, rendering such parameter sets inconsistent.

The unspecific affinity of the preleukemic cells is decreased in all consistent parameter sets (i.e. *u*^(^*^p^*^)^ < *u*^(*h*)^). This reduction generates a disadvantage of preleukemic species for accessing resources from non-preferred niches, thus prohibiting a dominating expansion. Nonetheless, the preleukemic species must have at least some residual unspecific affinity in order to generate results that are consistent with the experimental situation. Specifically, the residual unspecific affinity allows them to access resources from the unprotected niches in the monoclonal situation. Without this ability, these cells cannot outgrow the healthy species in this situation. When comparing the decrease of the specific and the unspecific affinity of the preleukemic cells (*u*^(^*^p^*^)^), it stands out that in scenarios P10 and P100, the specific affinity of the preleukemic cells (*s*^(^*^p^*^)^) is decreased to a greater degree than the unspecific affinity (*u*^(^*^p^*^)^) in all consistent parameter sets. In contrast, the decrease of the specific affinity of preleukemic cells (*s*^(^*^p^*^)^) is greater than their decrease in unspecific affinity (*u*^(^*^p^*^)^) in only ≈30% of the consistent parameter sets in scenario P1. Hence, if only a single preleukemic cell is present initially, the ability to receive a stimulus due to interaction with cognate self-peptide may be preserved or even increased, while in the situation of many initially present preleukemic cells the simulation results are not consistent with the experimental phenomena.

**Figure 6:**
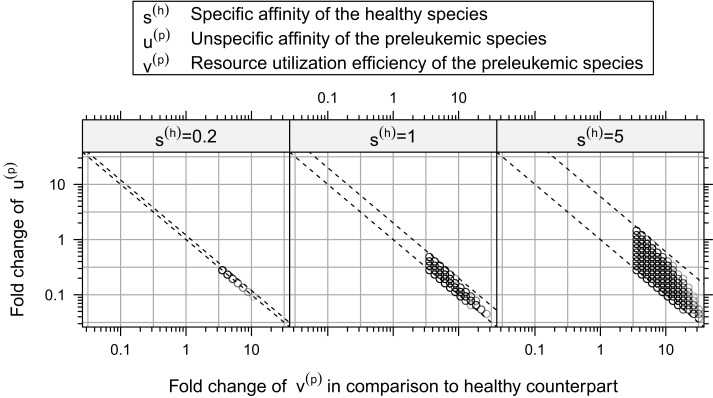
**Relation between resource utilization efficiency *v*^(*p*)^ and unspecific affinity *u*^(*p*)^ in the consistent parameter sets**. Here, the fold-changes of the unspecific affinities of the preleukemic cells *u*^(*t*)^ in the consistent parameter sets is plotted against their resource utilization efficiencies *v*^(*t*)^. The relation between these quantities is relatively strict. All points are contained between two parallel lines. In this plot, we show additional simulation results, in which also the specific affinity of the healthy cells *s*^(*h*)^ is varied.

Furthermore, our simulations demonstrate that there is a rather strict functional relation between the unspecific affinity of the preleukemic cells (*u*^(^*^p^*^)^) and their resource utilization efficiency (*v*^(^*^p^*^)^) for all consistent parameters (see Figure 6). In other words, a certain acquired growth advantage, which is mediated by an increase in resource utilization (*v*^(^*^p^*^)^), requires a corresponding decrease in the unspecific affinity (*u*^(^*^p^*^)^) to guarantee consistency. Interestingly, the stringency of this relation depends crucially on the specific affinity of the healthy cells (*s*^(*h*)^). To illustrate this relationship, we tested the system behavior also for *s*^(*h*)^ = 0.2 and *s*^(*h*)^ = 5 in addition to the value *s*^(*h*)^ = 1 used so far. Although varying values of *s*^(*h*)^ do not qualitatively change the system behavior, the size of the consistent parameter region, i.e. the number of consistent parameter sets, is affected considerably by the particular choice of *s*^(*h*)^: it is larger for *s*^(*h*)^ = 5 and smaller for *s*^(*h*)^ = 0.2. This can be seen in Figure 6, which illustrates the relationship for different values of *s*^(*h*)^.

Interpreting the specific affinity as affinity to cognate self-peptide, and the unspecific affinity as affinity to the MHC itself, the ratio of the specific and the unspecific affinity of the healthy cells (i.e. *s*^(h)/^*u*^(^*^h^*^)^) determines how strict the regulation exerted by the self-peptide defined niches is. If the ratio is large, the niche regulation is strict, i.e. T cells can hardly access resources from niches other than their preferred niche. If the ratio is small, T cells can acquire more resources from niches other than their preferred niche. Our simulation results indicate that the stronger the niche regulation (i.e. a larger *s*^(*h*)^), the larger is the consistent parameter region, and hence the more robust are the modeling results and the biological behavior of the system.

## 4 Discussion

Oncogenesis in mature T cells and especially the resistance to malignant transformation of these cells even in potent oncogenic transformation assays have raised substantial interest in these cell populations. In a series of experiments, it has been shown that the resistance of mature T cells depends on the diversity of the TCR repertoire. Specifically, MTCLLs could readily be induced in T-cell populations that are quasi-monoclonal with respect to their TCR, while oncogenesis occurred only in rare cases in the TCR polyclonal situation, despite identical experimental conditions (20, 21). This observation led to the hypothesis that in the polyclonal situation, regulation based on competition for survival stimuli mediated by interaction between TCRs and spMHCs on APCs can prevent the expansion of preleukemic cells. Furthermore, the hypothesis implies that this regulation fails in the quasi-monoclonal situation, and the preleukemic cells expand, and eventually give rise to overt malignancy.

We have developed and applied a mathematical model of T-cell homeostasis to qualitatively and quantitatively challenge this concept. In the model, mature T cells compete for resources that are provided by T-cell niches. Biologically, the resources correspond to survival stimuli from APCs due to interactions between spMHCs and the TCR. The different T-cell niches are defined by the different spMHCs found on APCs. Due to differential affinities of different TCRs to spMHCs, T cells may differ in their capability to acquire resources from a particular niche.

Within this modeling framework we systematically evaluated the effect of the model parameters. For each specific parameter set, we tested if it is consistent with the observed phenomena according to the criteria in Table 1.

In order to achieve consistency with the criteria, the niche affinities of the preleukemic cells had to be decreased (i.e. *s*^(^*^p^*^)^ < *s*^(*h*)^ and *u*^(^*^p^*^)^ < *u*^(*h*)^, while their resource utilization efficiency had to be increased (i.e. *v*^(^*^p^*^)^ > *v*^(*h*)^). Biologically, this suggests that the preleukemic cells are less dependent on niche resources (decreased affinities). In particular, our results predict a stronger decrease of the specific affinity of the preleukemic cells (*s*^(^*^p^*^)^) compared to the unspecific affinity (*u*^(^*^p^*^)^).

This implies that in the presence of healthy competitor cells with preference for the same niche, the preleukemic cells can be outcompeted. In the polyclonal situation this preventive effect acts on almost all niches, thus keeping the preleukemic cells at a minimum or even making them disappear.

In contrast, in the monoclonal situation the vast majority of niches is unguarded by healthy cells, and their resources can, therefore, be accessed by the preleukemic cells due to their albeit small, but non-zero unspecific affinity (*u*^(^*^p^*^)^). In this situation, the decrease in the affinities can be compensated by the increase of the resource utilization efficiency of the preleukemic cells (*v*^(^*^p^*^)^). Metaphorically speaking, the polyclonal healthy competitor cells protect the niche resources against the aggressive preleukemic cells in the polyclonal situation. If the polyclonal guards in the form of healthy competitor cells are not present, the preleukemic cells are able to claim all or most of the niche resources and promote MTCLL occurrence.

Transferring our findings back into a mechanistic context, we speculate that the decreased model affinities correspond to a down-regulation of TCR-mediated regulations and/or impairment of intracellular TCR signaling, i.e. a reduced T-cell avidity (25). A more efficient utilization of these signals (i.e. an increase in *v*^(*p*)^) can be interpreted as a partial independence of preleukemic cells from environment-mediated growth signals, or as failure to respond adequately to inhibitory signals. On an intracellular level this independence might be achieved by a constitutive up-regulation of growth-promoting pathways.

In order to describe the T-cell system, and in particular the experiments performed by Newrzela et al. in mathematical terms we made a number of simplifying assumptions. It is a long-standing notion that oncogenesis often develops in a multi-step process (26). Also, in the context of gene therapy, it has been described that oncogenesis may occur directly (*single hit induction*), or may require additional lesions (*cooperating hit induction*) (27, 28). In our simulation scenario, however, we consider the effect of a first but fully effective hit only, i.e. we assume a virtually instantaneous effect of the oncogene affecting all preleukemic cells after transduction. In principle, it would be possible to incorporate cooperating hits into our model that are subsequently acquired after transplantation. We refrained from doing so, since only preliminary experimental data on tumor evolution in the context of the phenomena in focus are available to date.

Similarly, we did not account for heterogeneity concerning the effect of the oncogene, i.e. all cells belonging to the preleukemic cell compartment are assigned the same functional alteration in the model. Also, it is not clear, if our model adequately describes the emergence of preleukemic cells on an individual clone level. Hopefully, such individual clone data will be available in the future, so that we will be able to validate our model in this regard.

In order to make numeric simulations feasible, we considered a system with only 100 niches and 100 T-cell clones in the polyclonal situation. This is several orders of magnitude below the estimated TCR diversity in mice, and the estimated number of self-peptides that can be presented per allele [∼10^5^ Ref. (13)]. However, there is significant cross-reactivity (i.e. presence of TCRs that may recognize more than one spMHC) in the T-cell system (29), so that the number of functionally relevant, distinct niches may be considerably lower. Being well aware of these simplifications, we consider the proposed system dimensions as sufficient in order to capture the general niche-based structure of the mature T-cell system.

Although we successfully demonstrated that suppression of preleukemic T cells due to TCR-related regulation in the polyclonal situation can consistently explain the experimental observations, we are aware that alternative explanations could also account for the observed phenomena. Definitive proof or disproof of the niche regulation hypothesis will require further experimental work.

Therefore, our conceptual understanding of T-cell oncogenesis will be challenged in further experiments. Specifically, we suggest to compare the phenotype of the leukemic/preleukemic cells with the phenotype of the healthy competitors regarding, e.g. their gene expression profile, their activation of relevant signaling pathways and their functional properties (30). This will allow assessing whether our assertions about the properties of the preleukemic cells (i.e. down-regulation of the TCR/TCR signaling and up-regulation of growth-promoting pathways) are correct. Our modeling results predict that there is a minimum clonal diversity that is needed in order to control preleukemic T cells as produced in the previous experiments. Further transplantation experiments are planned to determine the minimum TCR diversity in clonality titration experiments.

All simulations presented in this publication start with a hypocellular condition. However, additional simulations (data not shown) demonstrated that the model results are generally not dependent on the initial abundance of a species, as long as it is present at all. Therefore, our results apply also to leukemogenesis with physiological onset conditions as it occurs in clinical settings. The situation in which the malignancy develops from a single mutated cell (as can be expected for the majority of patients) corresponds to the presented scenario P1.

Central to this work is our assumption that the abundances of individual TCR-defined clones are regulated on a per-clone basis due to interactions with spMHCs. However, many aspects of the insinuated regulation are elusive to date. This is at least partially due to the fact that in the *in vivo* situation, accurate and precise time-dependent quantification of individual TCR-defined clones with abundances in the physiological range is technologically challenging, if not impossible, to date. Nonetheless, such a concept is intellectually appealing, and seems plausible on a mechanistic level. We hypothesize that this regulation is (at least in part) responsible for the relatively low incidence of mature T-cell malignancies relative to mature B-cell malignancies. This hypothesis would imply less effective control mechanisms in the mature B-cell system, which might be caused by the fact that clonal homeostasis in the mature B-cell system is presumably more complex than in the T-cell system, due to affinity maturation of the B-cell receptor, and ongoing influx of newly generated mature B-cell clones from the bone marrow. These complicating factors may undermine the effectiveness of the leukemia control mechanisms proposed for the mature T-cell system in the mature B-cell system.

So far in this publication, the unspecific affinities *u*^(*h*)^ and *u*^(^*^p^*^)^ are interpreted in terms of TCR affinity for the MHC molecule itself. However, these unspecific affinities could also represent the reliance of T-cell survival on survival cytokines, e.g. interleukin 7. Further studies could aim to disentangle these two potential components of the unspecific affinity. To do so, the use of mouse models with a restricted MHC repertoire might be useful.

As exemplified in this paper, mathematical modeling approaches allow for the quantitative assessment of functional principles of T-cell interactions, their integration into a consistent conceptual framework and the derivation of testable hypotheses. Our results add a new puzzle piece to the complex picture of mature T-cell homeostasis. The hypothesis that regulation based on TCR-defined clonal membership suppresses MTCLL emergence may serve as a novel starting point to delve deeper into the mechanisms governing the homeostatic behavior of mature T cells.

Our modeling results prompt further experimental research to clarify the nature of the differential transformability of mature T cells. Moreover, our work demonstrates the general ability of theoretical approaches to formalize and conceptually validate the results of experimental research and promotes the idea of an iterative, interdisciplinary approach to research in immunology.

## 5 Authors Contribution

Sebastian Gerdes performed mathematical modeling and wrote the paper, Sebastian Newrzela provided conceptual input and wrote the paper, Ingmar Glauche performed mathematical modeling and wrote the paper, Martin-Leo Hansmann provided conceptual input, Dorothee von Laer provided conceptual input, Ingo Roeder wrote the paper, provided conceptual input, and guided research.

## Conflict of Interest Statement

The authors declare that the research was conducted in the absence of any commercial or financial relationships that could be construed as a potential conflict of interest.

## References

[1] Casrouge A Beaudoing E Dalle S Pannetier C Kanellopoulos J Kourilsky P Size estimate of the αßTCR repertoire of naive mouse splenocytes. J Immunol (2000) 164(11):5782–7 10820256 10.4049/jimmunol.164.11.5782

[2] Naylor K Li G Vallejo AN Lee W-W Koetz K Bryl E The influence of age on T cell generation and TCR diversity. J Immunol (2005) 174(11):7446–52 15905594 10.4049/jimmunol.174.11.7446

[3] Jung D Alt FW Unraveling V(D)J recombination: insights into gene regulation. Cell (2004) 116(2):299–311 10.1016/S0092-8674(04)00039-X 14744439

[4] Huesmann M Scott B Kisielow P von Boehmer H Kinetics and efficacy of positive selection in the thymus of normal and T cell receptor transgenic mice. Cell (1991) 66(3):533–40 10.1016/0092-8674(81)90016-7 1868548

[5] Minter LM Osborne BA Cell death in the thymus – it’ s all a matter of contacts. Semin Immunol (2003) 15(3):135–44 10.1016/S1044-5323(03)00029-0 14563112

[6] Zal T Volkmann A Stockinger B Mechanisms of tolerance induction in major histocompatibility complex class II-restricted T cells specific for a blood-borne self-antigen. J Exp Med (1994) 180(6):2089–99 10.1084/jem.180.6.2089 7964486 PMC2191800

[7] Freitas AA Rocha B Population biology of lymphocytes: the flight for survival. Annu Rev Immunol (2000) 18(1):83–111 10.1146/annurev.immunol.18.1.83 10837053

[8] Singh NJ Bando JK Schwartz RH Subsets of nonclonal neighboring CD4+ T cells specifically regulate the frequency of individual antigen-reactive T cells. Immunity (2012) 37(4):735–46 10.1016/j.immuni.2012.08.008 23021952 PMC3478444

[9] Brocker T Survival of mature CD4 T lymphocytes is dependent on major histocompatibility complex class II-expressing dendritic cells. J Exp Med (1997) 186(8):1223–32 10.1084/jem.186.8.1223 9334361 PMC2199085

[10] Kirberg J Berns A von Boehmer H Peripheral T cell survival requires continual ligation of the T cell receptor to major histocompatibility complex-encoded molecules. J Exp Med (1997) 186(8):1269–75 10.1084/jem.186.8.1269 9334366 PMC2199081

[11] Martin B Bécourt C Bienvenu B Lucas B Self-recognition is crucial for maintaining the peripheral CD4+ T-cell pool in a nonlymphopenic environment. Blood (2006) 108(1):270–7 10.1182/blood-2006-01-0017 16527889

[12] Hataye J Moon JJ Khoruts A Reilly C Jenkins MK Naive and memory CD4+ T cell survival controlled by clonal abundance. Science (2006) 312(5770):114–6 10.1126/science.1124228 16513943

[13] Mahajan VS Leskov IB Chen JZ Homeostasis of T cell diversity. Cell Mol Immunol (2005) 2(1):1–10 16212905

[14] Morton LM Wang SS Devesa SS Hartge P Weisenburger DD Linet MS Lymphoma incidence patterns by WHO subtype in the United States, 1992-2001. Blood (2006) 107(1):265–76 10.1182/blood-2005-06-2508 16150940 PMC1895348

[15] Deeks S Wagner B Anton P Mitsuyasu R Scadden D Haung C A phase II randomized study of HIV-specific T-cell gene therapy in subjects with undetectable plasma viremia on combination antiretroviral therapy. Mol Ther (2002) 5(6):788–97 10.1006/mthe.2002.0611 12027564

[16] Recchia A Bonini C Magnani Z Urbinati F Sartori D Muraro S Retroviral vector integration deregulates gene expression but has no consequence on the biology and function of transplanted T cells. Proc Natl Acad Sci U S A (2006) 103(5):1457–62 10.1073/pnas.0507496103 16432223 PMC1360534

[17] Scholler J Brady TL Binder-Scholl G Hwang W-T Plesa G Hege KM Decade-long safety and function of retroviral-modified chimeric antigen receptor T cells. Sci Transl Med (2012) 4(132):ra53–132 10.1126/scitranslmed.3003761 22553251 PMC4368443

[18] Hacein-Bey-Abina S Von Kalle C Schmidt M McCormack M Wulffraat N Leboulch P LMO2-associated clonal T cell proliferation in two patients after gene therapy for SCID-X1. Science (2003) 302(5644):415–9 10.1126/science.1088547 14564000

[19] Ott M Schmidt M Schwarzwaelder K Stein S Siler U Koehl U Correction of X-linked chronic granulomatous disease by gene therapy, augmented by insertional activation of MDS1-EVI1, PRDM16 or SETBP1. Nat Med (2006) 12(4):401–9 10.1038/nm1393 16582916

[20] Newrzela S Cornils K Li Z Baum C Brugman MH Hartmann M Resistance of mature T cells to oncogene transformation. Blood (2008) 112(6):2278–86 10.1182/blood-2007-12-128751 18566328

[21] Newrzela S Al-Ghaili N Heinrich T Petkova M Hartmann S Rengstl B T-cell receptor diversity prevents T-cell lymphoma development. Leukemia (2012) 26(12):2499–507 10.1038/leu.2012.142 22643706

[22] De Boer RJ Perelson AS T cell repertoires and competitive exclusion. J Theor Biol (1994) 169(4):375–90 10.1006/jtbi.1994.1160 7967629

[23] Doherty P Riberdy J Belz G Quantitative analysis of the CD8+ T-cell response to readily eliminated and persistent viruses. Philos Trans R Soc Lond B Biol Sci (2000) 355(1400):1093–101 10.1098/rstb.2000.0647 11186311 PMC1692813

[24] De Boer RJ Perelson AS Ribeiro RM Modelling deuterium labelling of lymphocytes with temporal and/or kinetic heterogeneity. J R Soc Interface (2012) 9(74):2191–200 10.1098/rsif.2012.0149 22513720 PMC3405764

[25] McKee MD Roszkowski JJ Nishimura MI T cell avidity and tumor recognition: implications and therapeutic strategies. J Transl Med (2005) 3(1):35 10.1186/1479-5876-3-35 16174302 PMC1262785

[26] Rous P Beard J The progression to carcinoma of virus-induced rabbit papillomas (shope). J Exp Med (1935) 62(4):523–48 10.1084/jem.62.4.523 19870432 PMC2133298

[27] Baum C von Kalle C Staal FJ Li Z Fehse B Schmidt M Chance or necessity? Insertional mutagenesis in gene therapy and its consequences. Mol Ther (2004) 9(1):5–13 10.1016/j.ymthe.2003.10.013 14741772

[28] Fehse B Roeder I Insertional mutagenesis and clonal dominance: biological and statistical considerations. Gene Ther (2007) 15(2):143–53 10.1038/sj.gt.3303052 17972922

[29] Yin Y Mariuzza RA The multiple mechanisms of T cell receptor cross-reactivity. Immunity (2009) 31(6):849–51 10.1016/j.immuni.2009.12.002 20064442

[30] Wu S Jin L Vence L Radvanyi LG Development and application of phosphoflow as a tool for immunomonitoring. Expert Rev Vaccines (2010) 9(6):631–43 10.1586/erv.10.59 20518718 PMC2933839

